# Supplementary figures and images for: The Complex FtBBX22 and FtHY5 Positively Regulates Light-Induced Anthocyanin Accumulation by Activating FtMYB42 in Tartary Buckwheat Sprouts
Source: Int J Mol Sci. 2024 Jul 31;25(15):8376. doi: 10.3390/ijms25158376 (PMC11313212; doi:10.3390/ijms25158376)

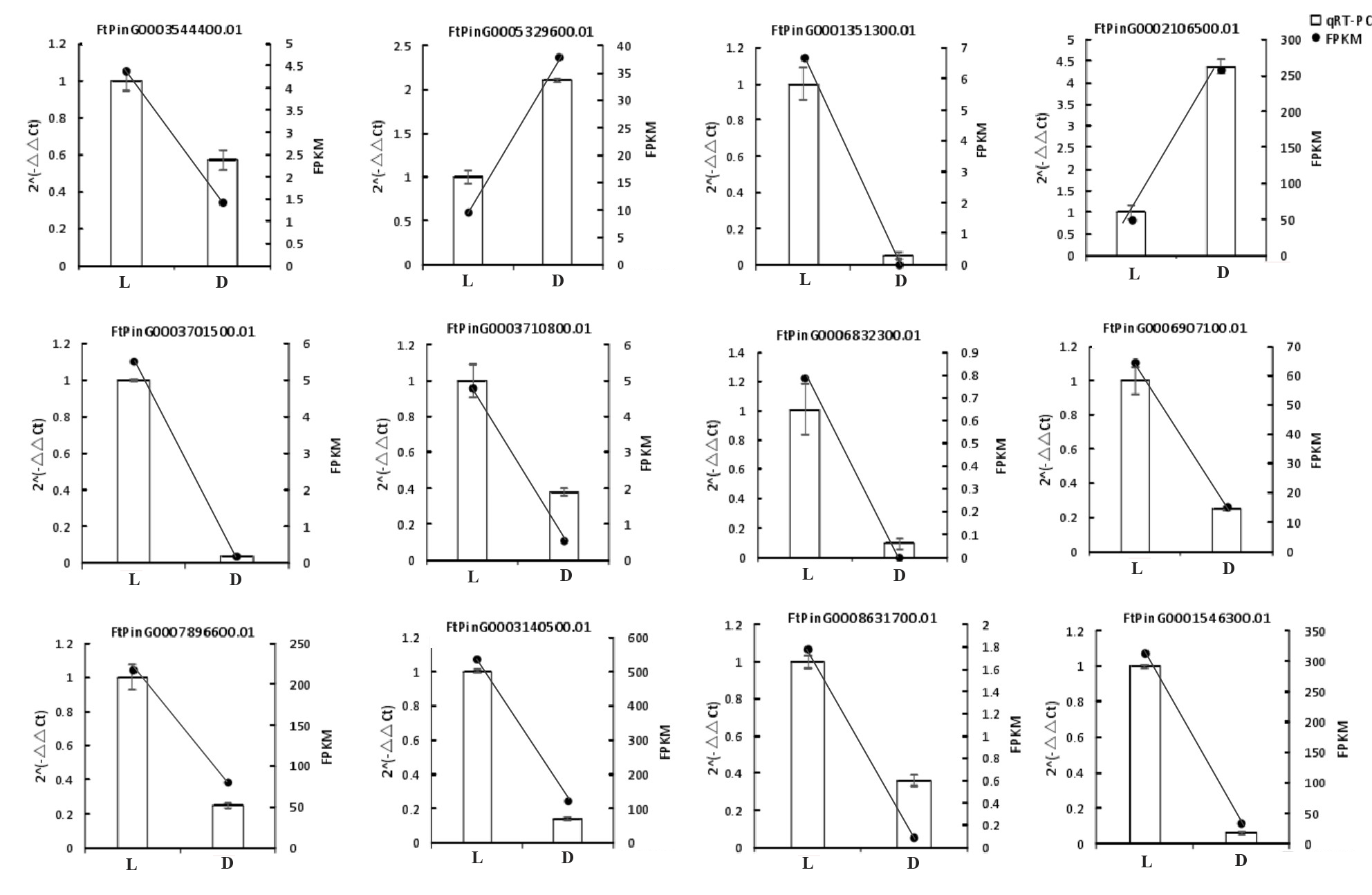

Supplement: Supplementary file 1 [file ijms-25-08376-s001.zip › Fig. S1.tif]
